# Silane-Coating Strategy for Titanium Functionalization Does Not Impair Osteogenesis *In Vivo*

**DOI:** 10.3390/ma14071814

**Published:** 2021-04-06

**Authors:** Plinio Mendes Senna, Carlos Fernando de Almeida Barros Mourão, Rafael Coutinho Mello-Machado, Kayvon Javid, Pietro Montemezzi, Altair Antoninha Del Bel Cury, Luiz Meirelles

**Affiliations:** 1 Health Science Center, Unigranrio University, Rio de Janeiro 25071-202, Brazil; 2 Biotechnology Department, Universidade Federal Fluminense, Niteroi 24070-035, Brazil; 3 Graduate Program, School of Dentistry, Universidade Federal Fluminense, Niteroi 24020-140, Brazil; rafaelcoutinhodemello@yahoo.com.br (R.C.M.-M.); onecure@aol.com (K.J.); 4 Private Practice, 24128 Bergamo, Italy; m.montemezzi@libero.it; 5 Department of Prosthodontics and Periodontology, Piracicaba Dental School, State University of Campinas, Piracicaba 13414-903, Brazil; altcury@fop.unicamp.br; 6 Department of Restorative and Prosthetic Dentistry, College of Dentistry, Ohio State University, Columbus, OH 43210, USA; luizmeirelles@yahoo.com

**Keywords:** titanium, osteogenesis, dental implants, implant surface, animal study

## Abstract

Silane-coating strategy has been used to bind biological compounds to the titanium surface, thereby making implant devices biologically active. However, it has not been determined if the presence of the silane coating itself is biocompatible to osseointegration. The aim of the present study was to evaluate if silane-coating affects bone formation on titanium using a rabbit model. For this, titanium screw implants (3.75 by 6 mm) were hydroxylated in a solution of H_2_SO_4_/30% H_2_O_2_ for 4 h before silane-coating with 3-aminopropyltriethoxysilane (APTES). A parallel set of titanium screws underwent only the hydroxylation process to present similar acid-etched topography as a control. The presence of the silane on the surface was checked by x-ray photoelectron spectroscopy (XPS), with scanning electron microscopy (SEM) and atomic force microscopy (AFM). A total of 40 titanium screws were implanted in the tibia of ten New Zealand rabbits in order to evaluate bone-to-implant contact (BIC) after 3 weeks and 6 weeks of healing. Silane-coated surface presented higher nitrogen content in the XPS analysis, while micro- and nano-topography of the surface remained unaffected. No difference between the groups was observed after 3 and 6 weeks of healing (*p* > 0.05, independent *t*-test), although an increase in BIC occurred over time. These results indicate that silanization of a titanium surface with APTES did not impair the bone formation, indicating that this can be a reliable tool to anchor osteogenic molecules on the surface of implant devices.

## 1. Introduction

Titanium has been widely used as an implant material due to its excellent corrosion resistance, biocompatibility and good mechanical properties such as high tensile strength, high ductility and low density [1]. However, osseointegration develops more often because of implant primary stability than from its degree of contact with the bone tissue [2]. Thus, to reduce early implant failures or complications during healing, especially for patients that present with metabolic diseases such as diabetes, osteoporosis and dental implants in immunocompromised patients [3,4,5,6,7] it is essential to modify the surface properties of titanium in an attempt to promote bone growth and to enhance the direct apposition of new bone in the early stages of the post-implantation period [8,9,10,11].

Therefore, an implant should allow surface regulation via cellular early-stage attachment for fast reconstruction of the primary implant stability [2,12], which would improve its short and, most importantly, its long-term performance [12]. In this context, the ability to immobilize bioactive molecules onto the titanium surface to create specific cellular responses is of great interest. Since covalently-bonded molecules exhibit greater stability on a surface than physisorbed compounds [13,14,15], especially considering the abrasion of the surface against bone during the implant insertion procedure [16,17], several strategies to chemically modify a titanium surface for the covalent bonding of biological molecules have been described, especially using silane chemistry [18,19,20].

One particular aminosilane, 3-aminopropyltriethoxysilane (APTES), is frequently used for bioconjugation techniques due to its bifunctional nature. APTES has three alkoxy groups that can attach to titanium hydroxyl groups via siloxane bonds; and on the other side of the molecule, nucleophilic amine groups serve as anchor points for further bonding of bioactive compounds of interest using crosslinking methods [21,22]. Enhanced cell bioactivity has been reported following the coupling of attachment of arginine-glycine-aspartic (RGD) acid or collagen onto titanium surfaces via an APTES coat in vitro [2,23,24,25,26]. However, in vivo studies to evaluate the use of an APTES coat on dental or orthopedic implants have not been conducted.

For bone healing to occur, numerous cellular and extracellular events take place at the implant/tissue interface. Given that siloxane bonds between APTES and titanium have been shown to be prone to hydrolysis in an aqueous environment [27,28], such as that of blood plasma, degradation of a silane layer could potentially affect the osseointegration of titanium implants. Thus, the present study aimed to investigate bone formation on an APTES-coated titanium surface using a rabbit model.

## 2. Materials and Methods

### 2.1. Silanization

Dental implants with titanium grade 4, size 3.75 by 6 mm, external hexagon platform (P-I Brånemark, São Paulo, Brazil) were hydroxylated in a solution of H_2_SO_4_/30%H_2_O_2_ (dilution ratio 1:1) for 1 h prior to being silanized by dipping in a 10% APTES (Sigma-Aldrich Corp., St. Louis, MO, USA) in boiling anhydrous toluene (Merck SA, Rio de Janeiro, Brazil) for 4 h under reflux [8,19]. It is critical to use an anhydrous solvent to control the increase the aminosilane polymerization that occurs on the surface [29]. Besides, the hydrogen bonds can be disturbed with high temperatures which then can reduce the number of infirm bonded silane molecules [30].

The titanium implants were subsequently cleaned in toluene and acetone (Merck SA/ Brazil, Rio de Janeiro, Brazil) during 10 min each in an ultrasonic bath to remove unbonded molecules. The implants were dried overnight at 110 °C in a vacuum oven to condensate the hydrogen-bonded silanols into siloxane bonds [29]. The control group only received titanium screws treated with acid (Ti) to present the same surface topography as the test group. All implants were sterilized with 25 kGy gamma radiation before use (CBE Embrarad, SP, Brazil).

### 2.2. Surface Morphology

The characterization of the surface was made by scanning electron microscopy (SEM) (Zeiss Auriga SEM/FIB, Oberkochen, Germany) at 1500× and 100,000× magnification. Surface topography was evaluated by atomic force microscopy (AFM) (Dimension Edge, Veeco Billerica, MA, USA) using a 125 nm cantilever in tapping mode (Digital Instruments, Santa Barbara, CA, USA). The pictures were digitally processed (Scanning Probe Image Processor software v.5.1.8; Image Metrology A/S, Hørsholm, Denmark) to calculate the roughness parameters, including the average height deviation (Sa), the developed interfacial area ratio (Sdr) and the summit density (Sds). By using X-ray photoelectron spectroscopy (XPS) (IFGW; Unicamp, Campinas, Brazil) under 5 × 10^−10^ torr with monochromatic (Al Kα) X-ray radiation as the active source, the chemical composition of each surface was determined.

### 2.3. Bone Formation Analysis

The Ethical Committee approved the present study by the protocol number 101443/2012-006, University of Rochester, NY, USA. Moreover, this research was described according to the ARRIVE checklist for the reporting of in vivo experiments. The rabbits were maintained throughout the experimental period in separate cages under conditions of controlled temperature (20 ± 2 °C) and humidity (55% ± 10%). The light-dark-cycle was 12:12 h, and the animals had free access to tap water and a standard pellet rabbit diet.

The sample size was calculated using a priori power analysis based on a previous study that evaluated bone-to-implant contact (BIC) in a similar rabbit model to estimate the effect size [31]. Considering type 1 error of 0.05 and power of 0.8, the two-tailed *t*-test determined a minimum of 9 implants in each group (G*Power 3.1.9.7; Universität Düsseldorf, Düsseldorf, Germany). Given expected attrition or death of animals of 10%, the sample size was defined as 10 implants per group/time point. To reduce the number of animals in the study, 2 implants were randomly applied to each proximal tibia metaphysis for a total of 40 implants inserted in 10 white rabbits, New Zealand race, 9 months old and weighting 3.5–4.0 kg.

Before the insertion of the implants, the rabbits received general anesthesia (0.3 mg/mL intramuscular fentanyl and 10 mg/mL fluanisone followed by 2.5 mg intraperitoneal diazepam). Each of the rabbit legs were shaved and treated with disinfectant, chlorhexidine prior to local anesthesia using 1 mL lidocaine at each insertion site. Skin and muscle layers were flapped, and drilling was performed on the bone at low speed with using abundant saline for cooling purposes. After dental implant placement, the fascia layers were sutured using resorbable sutures, and the skin was closed with polypropylene. After the surgery, all animals were transferred to appropriate housing with food and water ad libitum.

After 3 weeks and 6 weeks of healing, the rabbits were sacrificed with an overdose of pentobarbital (100 mg/kg) after sedation. All implants and the bone surrounding them were removed and fixed with 4% neutral buffered formaldehyde. Upon dehydration in a graded series of ethanols, the samples were embedded in light-curing resin (Technovit 7200 VLC, Heraeus Kulzer GmbH, Wehrheim, Germany). A histological section of each implant’s midline, 15 μm thick (equivalent to two cell layers), was prepared using a cutting and grinding system (Exakt; Norderstedt, Germany).

After the histological sections were stained with toluidine blue, BIC values were measured with a microscope (80i; Nikon Instruments, New York, NY, USA) using an imaging software (NIS-Elements BR 3.2, Nikon, New York, NY, USA) by a blinded examiner. BIC data were analyzed with an independent *t*-test at a significance level of 5%.

## 3. Results

**Figure 1:**
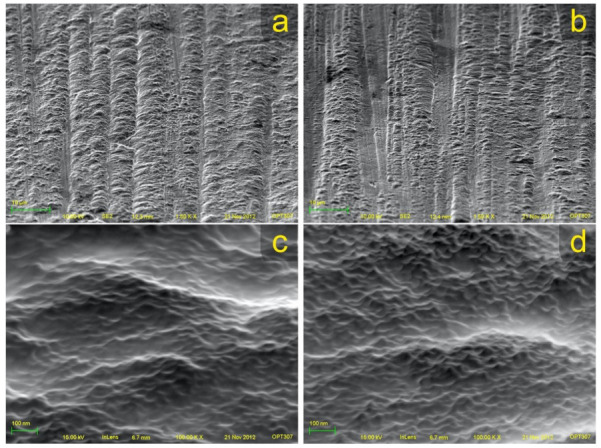
SEM images of the Ti surface (**a**,**c**) and the APTES-coated surface (**b**,**d**) showed similar surface morphology (1500× and 100,000× magnifications).

**Figure 2:**
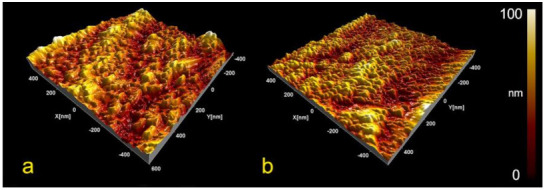
Three-dimensional reconstructions of the atomic force microscopy (AFM) data for the Ti (**a**) and APTES-coated (**b**) titanium surfaces. Similar nanostructure patterns were observed for both sets of screws.

**Figure 3:**
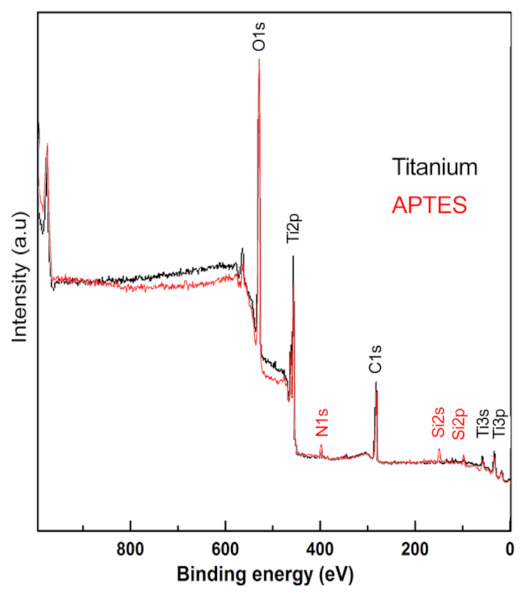
XPS spectra of the Ti (black line) and APTES-coated (red line) surfaces.

The titanium screw surfaces that were coated with APTES did not show any changes in surface morphology or roughness (Figure 1 and Figure 2). In addition, the SEM images of the APTES-coated screws showed a similar nanostructure pattern as the Ti screws. However, in the XPS spectra, small nitrogen (N 1s) and silicon peaks (Si 2s and Si 2p) were detected on the APTES-coated and not on the Ti surfaces, which indicated the presence of an APTES monolayer (Figure 3). The primary XPS region of a titanium surface is the titanium (Ti 2p) peak identified at approximately 458.6 eV, the expected location reported in the literature [32]. The oxygen (O 1s) peak is mainly from the titanium oxides but is enhanced by the hydroxylation procedure, where the OH^−^ acted as bound water [33]. In the APTES groups, the silane molecule has three atoms of oxygen for each nitrogen and silicon atom, contributing to the O 1s peak.

**Figure 4:**
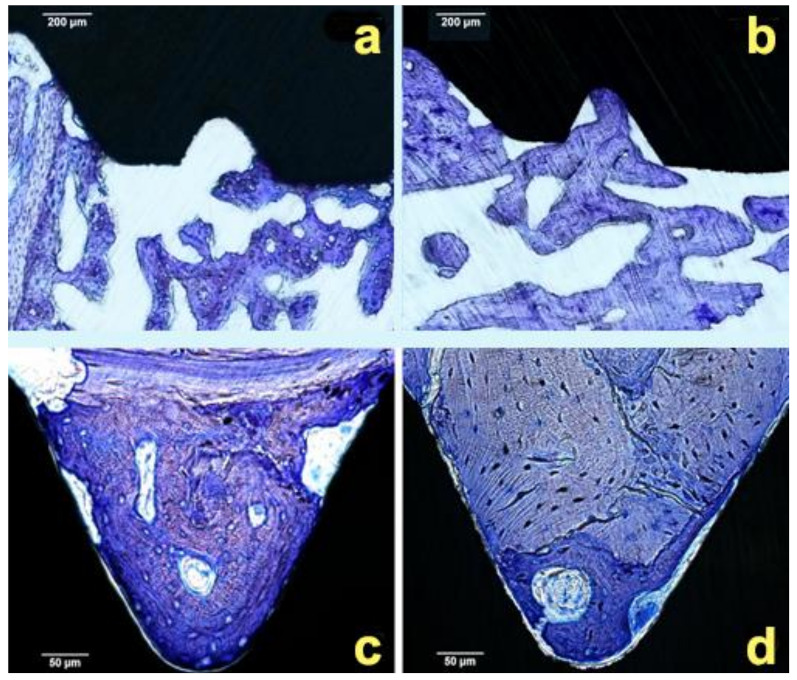
Bone healing proximal to the Ti (**a**) and APTES-coated (**b**) titanium implants (5× magnification) after 3 weeks of healing. After 6 weeks, the area inside threads were fulfilled with new bone formation Ti (**c**) and APTES-coated (**d**) titanium implants (20× magnification).

**Figure 5:**
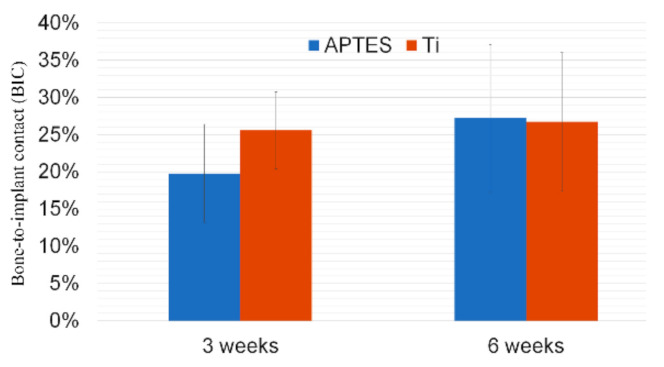
Bone-to-implant contact (BIC) values determined for the Ti and APTES-coated titanium implants after 3 weeks and 6 weeks of recovery.

There was no inflammatory response or adverse reaction observed proximal to any of the implants (Figure 4). The implant site in the tibia was characterized by a cortical layer 1.5 mm deep. After 3 weeks, the implants at both sites exhibited a typical endosteal reaction leading to new bone growth from the cortical layer. Correspondingly, similar BIC values of 26% ± 5% and 20% ± 7% were detected for the Ti and APTES-coated implants, respectively (*p* > 0.05). After 6 weeks of healing, the newly formed mineralized tissues were found to contain osteocytes and osteoblasts, thus indicating that continuous mineralization of the tissues had occurred (Figure 5). The BIC values after 6 weeks were 27% ± 9% and 27% ± 10% for the Ti and APTES-coated implants, respectively (*p* > 0.05).

## 4. Discussion

Typically, when bone formation is evaluated before the clinical use of a surface in humans, the tibia and femur of rabbits are the recommended models according to the International Organization for Standardization (ISO 10993-6:2007). These models were used in the present study. Our findings indicate that the coating of a titanium surface with APTES did not affect the morphological surface of the titanium implants tested, and it also did not impair early bone formation. Moreover, similar bone formation starting from the endosteum was observed in both the Ti and APTES-coated groups due to blood vessel disruption. After 6 weeks all the implants were found to be integrated with bone tissue.

Currently, dental implants were manufactured using commercially pure titanium (grade 2 or grade 4) or titanium alloys and receive surface treatments to improve bone formation [34] Although pure titanium is slightly favored over titanium alloys [35,36], grade 4 titanium was selected in the present study because of its improved mechanical performance [34]. To avoid the influence of surface topography on osseointegration [37], all the implants in the present study had the same design and were inserted using the same surgical protocol. Furthermore, the control group was acid-etched in the same manner as the APTES group, and both groups exhibited a minimally rough surface. Acid etching with hydrogen peroxide increases the number of hydroxyl groups that are available on a titanium surface for reaction with silane molecules. In addition, APTES have three alkoxy groups on each molecule to react with the surface, in which previous studies have reported an APTES coverage surface ranging between 0.25 nmol/cm^2^ to 5 nmol/cm^2^ on titanium [24,38,39]. No changes in the APTES layer were described when the temperature was below 50 °C [28], assuming that it was stable in body temperature. However, the possible mechanism for hydrolytic degradation needs to be investigated.

The histological findings of the present study demonstrated no inflammatory infiltrate, bone resorption or foreign body reaction in the periods evaluated, which corroborates titanium’s biocompatibility and indicates the biocompatibility of the APTES-coating. Although it was not statistically significant, the BIC values after 3 weeks of healing indicate lower values for the APTES-coated group, suggesting a slight delay in osseointegration in terms of bone-implant contact rates. However, at 6 weeks, bone formation was equivalent. Unfortunately, there was no other in vivo study for comparison. A previous in vitro study [28] reported lower adhesion of human osteosarcoma cells on the silanized titanium surface, which may explain a possible delayed bone formation. However, in this same study, greater adhesion than titanium was found when adhesion peptides were bonded on the silane layer. Thus, to enhance early bone formation, the attachment of osteogenic molecules should be an exciting strategy.

In the XPS spectra, the nitrogen peak that was detected indicated that nitrogen is primarily present as an –NH_2_ group on an APTES-coated surface. When surfaces undergo a curing step at high temperatures, this usually converts any protonated amines to neutral amines, thereby providing more versatility in subsequent applications [40]. However, these amino groups can also undergo protonation under physiological conditions, and this leads to a change in the surface charge from negative (isoelectric point (pI): 5.0–5.9) to favorable (pI: 8.5) based on the charge associated with titanium oxide versus amino-functionalized titanium [38,41]. This change in surface charge can also enhance the electrostatic interactions between a titanium surface and blood plasma proteins (pI < 7.4), thereby affecting the sequence of events that lead to bone formation [42]. Although protein adsorption was not evaluated in the present study, the APTES coating did not affect bone formation.

The main drawback of an APTES coating is the hydrolytic instability of the siloxane bond in an aqueous medium, such as blood, leading to a loss of covalently attached silane molecules [28,43]. As a limitation of the present study, a XPS analysis of the APTES-coated titanium surface is not possible after the 6 weeks in the bone tissue to investigate silane layer stability due to the contamination of the surface with bone. While most of the literature on aminosilanes has focused on reaction conditions for the preparation of covalently attached silane layers with controlled thickness and topography, the attached aminosilane layers’ hydrolytic stability is vital to the applications and further derivatizations of the functionalized substrates in aqueous media. In the present study, if any detrimental effects by the degradation of the APTES layer was present, it did not affect bone healing, possibly due to the slow-release rate of the silane molecules. However, future studies need to investigate bioactive material coatings’ stability on a surface following their implantation into bone [17].

## 5. Conclusions

Silanization of a titanium surface with APTES did not impair bone formation, indicating that this can be a reliable tool to anchor osteogenic molecules on implant devices’ surfaces.

## Data Availability

Not Applicable.
